# Long noncoding RNA MALAT1 knockdown reverses chemoresistance to temozolomide via promoting microRNA‐101 in glioblastoma

**DOI:** 10.1002/cam4.1384

**Published:** 2018-02-26

**Authors:** Tao Cai, Yu Liu, Jie Xiao

**Affiliations:** ^1^ Department of Neurosurgery The Third Xiangya Hospital Central South University 138 Tongzipo Road Changsha Hunan 410013 China; ^2^ Department of Emergency The Third Xiangya Hospital Central South University 138 Tongzipo Road Changsha Hunan 410013 China

**Keywords:** Chemoresistance, glioblastoma, LncRNA MALAT1, miR‐101, temozolomide

## Abstract

Glioblastoma (GBM) is the most common and lethal tumor of the central nervous system with highly infiltrative and resistant to chemotherapy. Temozolomide (TMZ) is widely used as the first‐line treatment for the therapy of GBM. However, a considerable percentage inherent or acquired resistance in GBM accounts for many treatment failures of the TMZ chemotherapy. Therefore, a deeper understanding of the molecular characteristics underlying TMZ resistance and the identification of novel therapeutic target is urgent. Here, we show that MALAT1 was significantly upregulated in TMZ‐resistant GBM cells. On the other hand, MALAT1 knockdown reduces TMZ resistance of GBM cells both in vitro and in vivo by inhibiting cell proliferation and promoting apoptosis. We also show that miR‐101 overexpression reduced TMZ resistance of GBM cells and played an antagonistic role compared with MALAT1. Importantly, we demonstrate that MALAT1 promoted the chemoresistance through suppressing miR‐101 signaling pathway via directly binding it in GBM cells. In conclusion, our study indicates that knockdown of MALAT1 reverses chemoresistance to TMZ via promoting miR‐101 regulatory network in GBM and thus offers a novel prognostic marker and potential target for GBM TMZ‐based chemotherapy.

## Introduction

Glioblastoma (GBM), also known as glioblastoma multiform, is the most common and lethal tumor of the central nervous system because it reproduces quickly and it is supported by a large network of blood vessels 1, 2. Temozolomide (TMZ), an alkylating agent which was developed by Malcolm Stevens et al., is an oral chemotherapy drug, and it is widely used as the first‐line treatment for GBM 3. However, a considerable percentage of GBMs have inherent or acquired resistance to TMZ‐based chemotherapy, which critically impedes the clinical outcome 4, 5. Various studies have been carried out to explore how GBM cells acquire resistance to TMZ; however, the underlying mechanisms remain largely unknown 6. Thus, a deeper understanding of the molecular characteristics underlying this resistance and the identification of novel therapeutic target is imperative.

Long noncoding RNAs (lncRNAs), generally longer than 200 nucleotides in length, are emerging as important regulators in tumor initiation and progression, as indicated by numerous studies in recent years 7, 8, 9. The key roles of lncRNAs played in drug resistance are also extensively reported 10, 11. For instance, Zhang et al. 12 reported that lncRNA ODRUL acted as a pro‐doxorubicin‐resistant molecule through inducing the expression of the classical multidrug resistance‐related ABCB1 gene in osteosarcoma cells. Li et al. 13 demonstrated that lncRNA MALAT1 was associated with poor response to oxaliplatin‐based chemotherapy in patients with colorectal cancer and promotes chemoresistance through EZH2. MicroRNAs (miRNAs) are a class of short, single‐stranded RNAs that regulate gene expression through either the inhibition of translation or mRNA degradation 14, 15. Recently, miRNAs have been indicated to be involved in the regulation of tumorigenesis, differentiation, and chemoresistance through the inhibition of major cellular pathways 16, 17, 18. For example, Tian et al. reported that miR‐101 reversed TMZ resistance by inhibition of GSK3*β* in GBM 19. Although some studies demonstrated the important roles of lncRNAs or miRNAs played in the regulation of chemoresistance, the association of specific lncRNA or miRNA with drug resistance in GBM cells is still largely unknown.

In this study, we applied quantitative reverse transcription‐PCR (qRT‐PCR) assay to assess the expression of lncRNA MALAT1 and miR‐101 in TMZ‐resistant GBM cell lines and the parental cell lines. In addition, functional studies of MALAT1 and miR‐101 were conducted to determine their potential roles in regulation of TMZ resistance. Furthermore, our data indicate a direct binding between MALAT1 and miR‐101, and this interaction played important function in regulation of chemoresistance in GBM.

## Materials and Methods

### Cell culture and reagents

The human GBM cell line U251 was purchased from Auragene Bioscience Inc., Changsha, China. The cells were cultured in Dulbecco's modified Eagle's medium (DMEM; Hyclone, USA) supplemented with 10% fetal bovine serum (FBS; Thermo Fisher, Waltham, MA), 100 U/mL penicillin, and 100 mg/mL streptomycin (Life Technologies, Carlsbad, CA). Cultures were maintained at 37°C in humidified air with 5% CO_2_. TMZ (S1237) was purchased from Selleck Chemicals.

### Establishment of TMZ‐resistant cell lines

U251 cells (1 × 10^5^/mL) were exposed to an initial TMZ concentration of 1 *μ*mol/L for 15 days. The surviving population of cells was grown to 80% confluence and passaged twice over 15–20 days. The concentration of TMZ was then sequentially increased in the same manner to 5 *μ*mol/L (15 days), 25 *μ*mol/L (15 days), 50 *μ*mol/L (15 days), 100 *μ*mol/L (20 days), 200 *μ*mol/L (20 days), and finally to the concentration of 400 *μ*mol/L (25 days) 20. The established TMZ‐resistant cell lines were designated as U251/TMZ.

### RNA extraction and qRT‐PCR analyses

Total RNA was extracted from frozen cells with the TRIzol reagent (Invitrogen, Shanghai, China), and the cDNA was synthesized using the Reverse Transcription Kit (Takara, China). The amount and quality of RNA were determined based on absorbance ratios with NanoDrop Lite (Thermo, USA). qRT‐PCR analyses were performed using SYBR Green qPCR Mix (TOYOBO, Osaka, Osaka Prefecture, Japan) according to the manufacturer's instructions. The expression levels of target genes were normalized to the transcription level of *β*‐actin. The data were collected and calculated using an ABI 7300 instrument (Life Tech). Primers for qRT‐PCR were synthesized by Invitrogen (Shanghai, China) and the sequences were: MALAT1 sense, 5′‐GACCCTTCACCCCTCACC‐3′ and antisense, 5′‐TTATGGATCATGCCCACAAG‐3′; miR‐101, HmiRQP0021; U6, HmiRQP9001; *β*‐actin sense, 5′‐AGGGGCCGGACTCGTCATACT‐3′; and antisense, 5′‐GGCGGCACCACCATGTACCCT‐3′.

### In situ hybridization analysis

The in situ hybridization (ISH) probe used for detecting MALAT1 was synthesized by Sangon Biotech (Shanghai, China). The probe sequence was designed as ACATTGCCTACCACTCTAAGA. Slices were processed using Enhanced Sensitive ISH Detection Kit I (Boster, Wuhan, Hubei, China) according to the manufacturer's instructions. Then, they were visualized with nitroblue tetrazolium chloride/5‐bromo‐4‐chloro‐3‐indolyl phosphate (Solarbio, Beijing, China) for 5 min and counterstained with nuclear fast red for 60 sec. And slides were photographed and quantitated with Olympus BX51 microscope (Olympus, Japan).

### Western blot analysis

Proteins were extracted from cells with RIPA lysis buffer (Auragene Bioscience), which was supplemented with a protease inhibitor cocktail (Auragene Bioscience) and PMSF (Auragene Bioscience). Equal amounts (10 *μ*g) of proteins were loaded on SDS‐PAGE and then were transferred to a PVDF Immobilon‐P membrane (Millipore, Billerica, MA). The membrane was blocked with 3% BSA‐TBST at room temperature for 90 min and continuously was probed with indicated primary antibodies at 4°C overnight. Then, the membranes were washed and incubated with specific secondary antibodies 1 h. A *β*‐actin antibody was used as a control, and the MRP1 (1:500; Abcam), P‐gp (1:400; Proteintech), MGMT (1:800; Abcam), cleaved‐PARP (1:800; Abcam), and GSK3*β* (1:1000; ImmunoWay) antibodies were used for each group.

### Cell transfection and lentivirus transduction

The miR‐101 mimic (HmiR‐AN0021‐SN), miR‐101 inhibitor (HmiR‐AN0021‐SN‐10), and relative controls were purchased from GeneCopoeia Inc., Guangzhou, Guangdong, China. The transfection of mimic, inhibitor, and related controls was carried out using Lipo6000^™^ (Beyotime, Nanjing, Jiangsu, China) according to the manufacturer's instructions. To suppress the expression of MALAT1, shMALAT1 sequence (target sequence: GACAGGTATCTCTTCGTTATC) was synthesized. The sequences were cloned into the BamHI and EcoRI sites of pGMLV‐SC5 with the forward oligo, gatccGACAGGTATCTCTTCGTTATC***TTCAAGAGA***GATAACGAAGAGATACCTGTCTTTTTTg and reverse oligo, aattcAAAAAAGACAGGTATCTCTTCGTTATC***TCTCTTGAA***GATAACGAAGAGATACCTGTCg. Lentiviral production, titration, and infection were performed as Li et al. described 21. Lentiviral plasmids pGMLV‐SC5 expressing shMALAT1 or control was cotransfected with the packaging vectors psPAX2 and pMD2.G into 293T cells using HG transgene reagent (Genomeditech, China). Lentiviral particles were harvested after 48 h of transfection. The U251/TMZ cells were then infected with lentiviruses and prepared for the further experiments. The expression of MALAT1 in cells was determined using qRT‐PCR.

### Cell proliferation assay

The cell proliferation was assessed using 3‐(4,5‐dimethylthiazol‐2‐yl)‐2,5‐diphenyltetrazolium bromide (MTT) solution (Sangon Biotech). Forty‐eight hours after transfection, the GBM cells were seeded into 96‐well plates at an initial density of 5 × 10^3^ cells/well. After 24 h of culture, these cells were exposed to 50 *μ*mol/L, 100 *μ*mol/L, 200 *μ*mol/L, 300 *μ*mol/L, and 400 *μ*mol/L TMZ for 24 h, respectively. Then, the cells were treated with 10 *μ*L MTT by adding it to each well. The cells were incubated at 37°C with 5% CO_2_ for another 4 h, then the medium was removed carefully, and 150 *μ*L dimethyl sulfoxide (DMSO) solution (MP Biomedicals, USA) was added for 10 min to lyse the cells. Subsequently, the absorbance was measured at 570 nm using a microplate reader Multiskan MK (Thermo Scientific, Waltham, MA). The survival rate was calculated using the equation: (mean absorbance of drug well/mean absorbance of control wells) × 100%.

### Colony formation assay

The GBM cells were placed into nine‐well plates and maintained in DMEM containing 10% FBS and 300 *μ*mol/L TMZ. After 2–3 weeks, cells were fixed with 4% methanol and stained with GIMSA for 10–30 min. The visible colonies were manually counted, and the rate of colony formation was calculated with the following equation: (number of colonies/number of seeded cells) × 100%.

### Flow cytometric analysis

The apoptosis rate was assessed by flow cytometric analysis. The harvested GBM cells were washed twice with cold phosphate‐buffered saline (PBS). Then, these cells were fixed with cold 70% ethanol overnight and stained with 5 *μ*L annexin V‐isothiocyanate (FITC) and 5 *μ*L PI (Keygentec, Nanjing, Jiangsu, China) for 5–15 min in the dark at room temperature. The cells were then examined by flow cytometry (BD Biosciences, Franklin Lakes, NJ).

### TdT‐mediated dUTP nick end labeling (TUNEL) assay

Apoptotic cells were identified using in situ cell death detection kit (Roche Applied Sciences, #1168479591; Penzberg, Upper Bavaria, Germany) according to the manufacturer's instructions. Nuclei were stained with DAPI. An inverted laser scanning confocal microscope at ×100 magnification (Zeiss, #710, Oberkochen, Baden Wurttemberg, Germany) was used for imaging, and ImageJ software was used for quantification of TUNEL‐positive cells.

### Tumor formation assay in a nude mouse model

Four‐week‐old male BALB/C‐NU mice were obtained from the Hunan SJA Laboratory Animals Center of the Chinese Academy of Sciences. The mice were housed under pathogen‐free conditions. To assess the tumor formation, the mice were injected with 2 × 10^7^/mL U251/TMZ cells subcutaneously. The weights of mice and tumor volumes were recorded every 3 days as the tumor reached 50–70 mm^3^. Tumor volume was calculated as follows: tumor volume = (width^2^×length)/2. At 25 days after U251/TMZ cells injection, it began to inject 5 mg/kg/day TMZ into the flank. After 35 days, the mice were killed, and the tumors derived from each group were used for comparison.

### Dual‐luciferase reporter assay

The fragments from MALAT1 containing the predicted miR‐101 binding site were synthesized and cloned into the luciferase construct psi‐CHECK2. The resulted vector MALAT1‐3′UTR‐psi‐CHECK2 was called the reporter vector MALAT1‐Wt. The corresponding mutant was called MALAT1‐Mut. The miR‐101 mimic or negative control miR‐NC was cotransfected with the reporter vectors using transfection reagent (Invitrogen, USA). Forty‐eight hours after transfection, firefly and renilla luciferase activities in cell lysates were measured using the Dual‐Luciferase Reporter Assay Kit (E1910; Promega, Madison, WI).

### Statistical analysis

Error bars in figures represent SD (Standard Deviation), from at least three independent experiments. The paired *t*‐test was used for statistical analyses between groups, as appropriate. *P *<* *0.05 was considered to be statistically significant. Statistical analysis was performed with the GraphPad Prism 5.0 (GraphPad Software, San Diego, CA).

## Results

### High expression of MALAT1 associates with TMZ resistance in GBM cells

**Figure 1:**
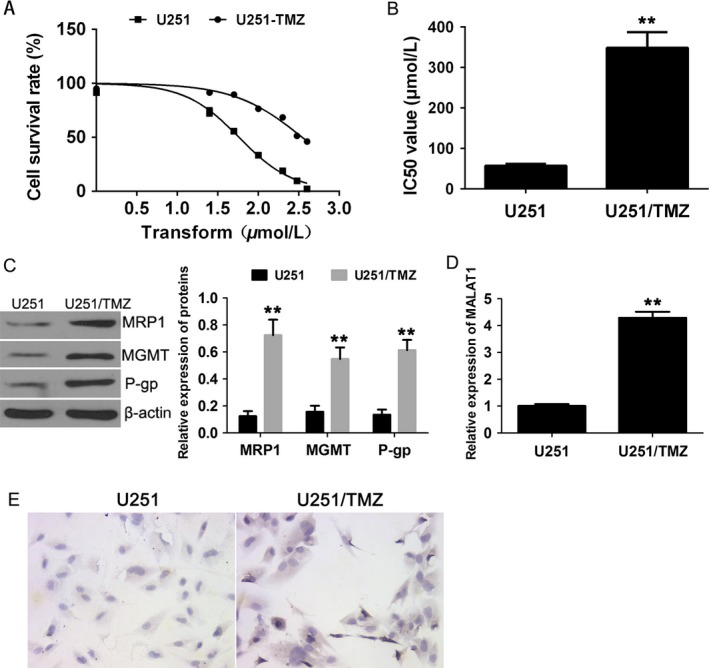
High expression of MALAT1 was associated with TMZ resistance in glioma cells. (A) Cell survival rates of GBM cell lines U251 and U251/TMZ were assessed by MTT assay. (B) The IC50 values of TMZ in U251 and U251/TMZ. (C) The expressions of MRP1, MGMT, and P‐gp were evaluated by Western blotting, and *β*‐actin was used as control. (D) The mRNA expressions of MALAT1 in cell lines U251 and U251/TMZ were assessed by qRT‐PCR analysis, and *β*‐actin was used as control. (E) Representative images of ISH for MALAT1 in U251 and U251/TMZ cells. All data represent the means ± SD, and all experiments were performed in triplicate. **P < 0.01.

To explore the role of MALAT1 in TMZ resistance of glioma cells, we first established TMZ‐resistant GBM cell line U251 as described in Materials and Methods. As shown in Figure 1A, the cell survival rate of TMZ‐resistant cell line U251/TMZ and the parental cell line declined in a TMZ (0‐400 *μ*mol/L) dose‐dependent manner, and U251/TMZ showed significantly improved resistance to TMZ compared with the parental cell lines. The half maximal inhibitory concentration (IC50) values of TMZ in U251 and U251/TMZ were (57.63 ± 2.1) *μ*mol/L and (348.8 ± 34.32) *μ*mol/L, respectively (Fig. 1B). In addition, we examined the expressions of chemoresistance‐related genes in these GBM cell lines, including multidrug resistance‐associated protein 1 (MRP1), O^6^‐methylguanine‐DNA methyltransferase (MGMT), and P‐glycoprotein (P‐gp). The U251/TMZ showed elevated expressions of these chemoresistance‐related genes, suggesting that changes of gene regulation network were involved in the TMZ resistance of U251/TMZ (Fig. 1C). Subsequently, we sought to determine whether MALAT1 was dysregulated in TMZ‐resistant cell line U251/TMZ. To address this question, we examined the mRNA expressions of MALAT1 in cell lines U251/TMZ and U251 using qRT‐PCR (Fig. 1D). The expression of MALAT1 increased significantly in U‐251/TMZ compared with the parental cell. Moreover, the ISH assay also indicated a high expression of MALAT1 correlates with TMZ resistance in GBM cells (Fig. 1E). And the results were supported by the study using GBM cell line U87 as well (Fig. S1).

### Knockdown of MALAT1 reduces chemoresistance in TMZ‐resistant GBM cells in vitro and in vivo

**Figure 2:**
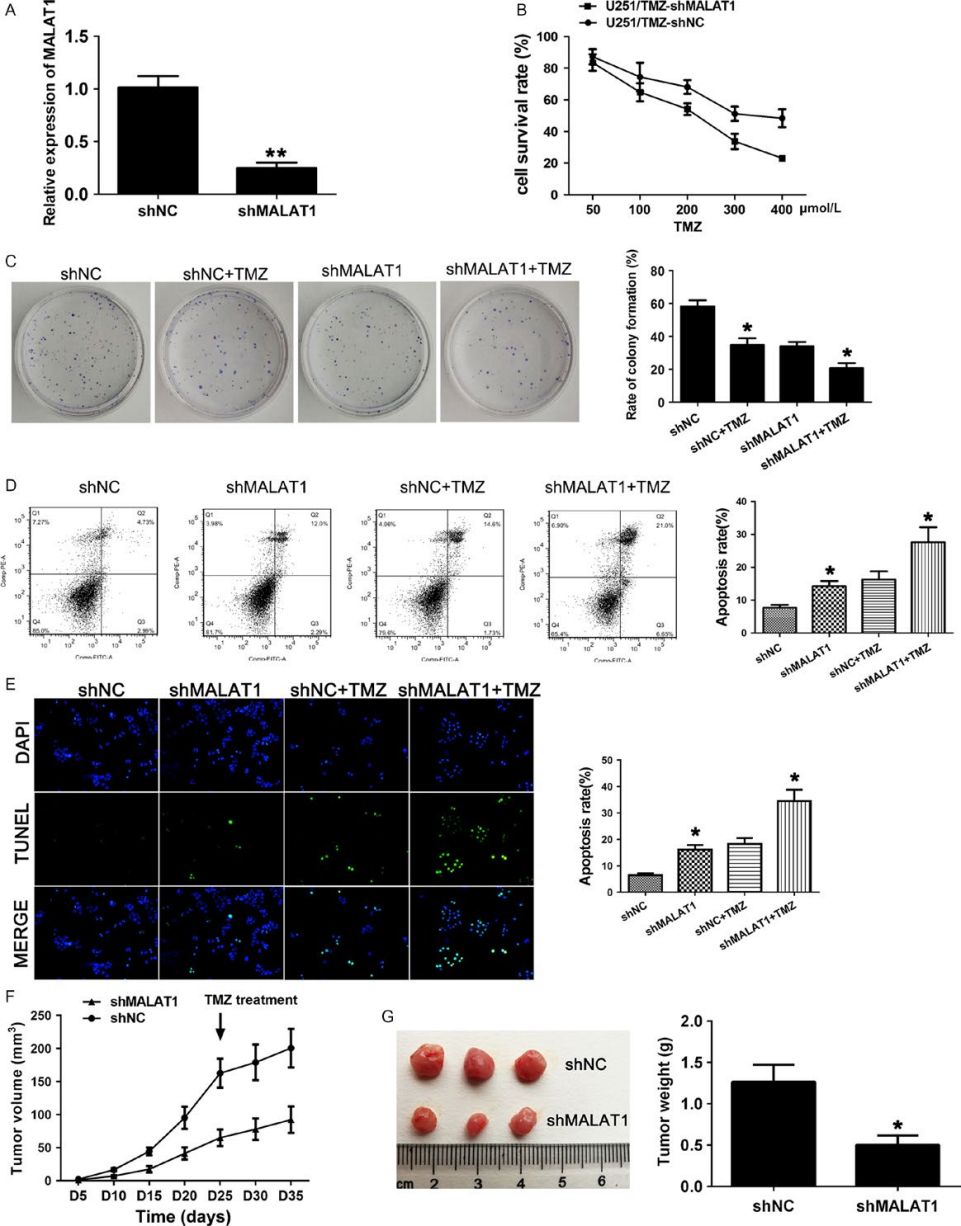
Knockdown of MALAT1 reduces chemoresistance in TMZ‐resistant GBM cells in vitro and in vivo. (A) The relative expressions of MALAT1 in the U251/TMZ cells transduced with shMALAT1 or shNC were determined by qRT‐PCR, and *β*‐actin was used as control. (B) The cell survival rates of transgenic cell lines with TMZ (50–400 *μ*mol/L) treatments were determined by MTT assay. (C) Colony formation assay showed the numbers of colonies of U251/TMZ cells transduced with shMALAT1 or shNC in the presence or absence of TMZ. (D) Annexin V/PI staining and flow cytometry analysis was used to assess apoptosis in GBM cell lines. (E) Representative DAPI‐stained nuclei and terminal deoxynucleotide transferase dUTP nick end labeling‐stained apoptotic nuclei of each group. (F) Tumor growth curve was based on the tumor volumes which were calculated every 5 days, and the TMZ treatment (5 mg/kg/day) began at 25th day after injection. (G) Tumors were harvested and measured at the 35th day after injection. All data represent the means ± SD of three replications. *P < 0.05, **P < 0.01.

As MALAT1 were highly upregulated in TMZ‐resistant GBM cell line U251/TMZ, we used MALAT1 knockdown GBM cell line U251/TMZ‐shMALAT1 to explore the role of MALAT1 played in TMZ resistance. The efficiency of MALAT1 knockdown was examined by qRT‐PCR analysis. The expression of MALAT1 in the U251/TMZ‐shMALAT1 was significantly suppressed as compared with the control group (Fig. 2A). To investigate whether the knockdown of MALAT1 in U251/TMZ‐shMALAT1 could influence the TMZ resistance, MTT assay was performed. We found the knockdown group U251/TMZ‐shMALAT1 showed decreased resistance to TMZ compared with control group (Fig. 2B). Besides, we performed colony formation assay. In the absence of TMZ, the U251/TMZ‐shMALAT1 showed a decreased rate of colony formation. In the presence of TMZ, knockdown of MALAT1 sensitized U251/TMZ cells to the treatment, as indicated by the significantly decreased rate of colony formation (Fig. 2C). To further determine whether decreased TMZ resistance of GBM reflected cell apoptosis, the flow cytometry assays and TUNEL assay were performed. The results showed that both TMZ‐treated or nontreated U251/TMZ‐shMALAT1 had higher apoptotic rates in comparison with the control group, and the apoptosis rate of shMALAT1 group was significantly increased with the treatment of TMZ (Fig. 2D and E). To evaluate the effect of MALAT1 in TMZ resistance in vivo, the U251/TMZ‐shMALAT1 or U251/TMZ‐shNC cells were injected into flanks of nude mice. Consistent with the in vitro results, tumor growth in the shMALAT1 group was significantly attenuated compared to the control group (Fig. 2F). Following the TMZ treatment began at 25th day after injection, knockdown of MALAT1 reduced the TMZ resistance of shMALAT1 group. Moreover, after tumors were harvested, both the tumor volume and weight in shMALAT1 group were significantly reduced compared to the control group. And the results were supported by the study using GBM cell line U87 as well (Fig. S2). Taken together, our results indicated that low expression of MALAT1 reversed chemoresistance in TMZ‐resistant GBM cells both in vitro and in vivo (Fig. 2G).

### MALAT1 is a direct target of miR‐101

**Figure 3:**
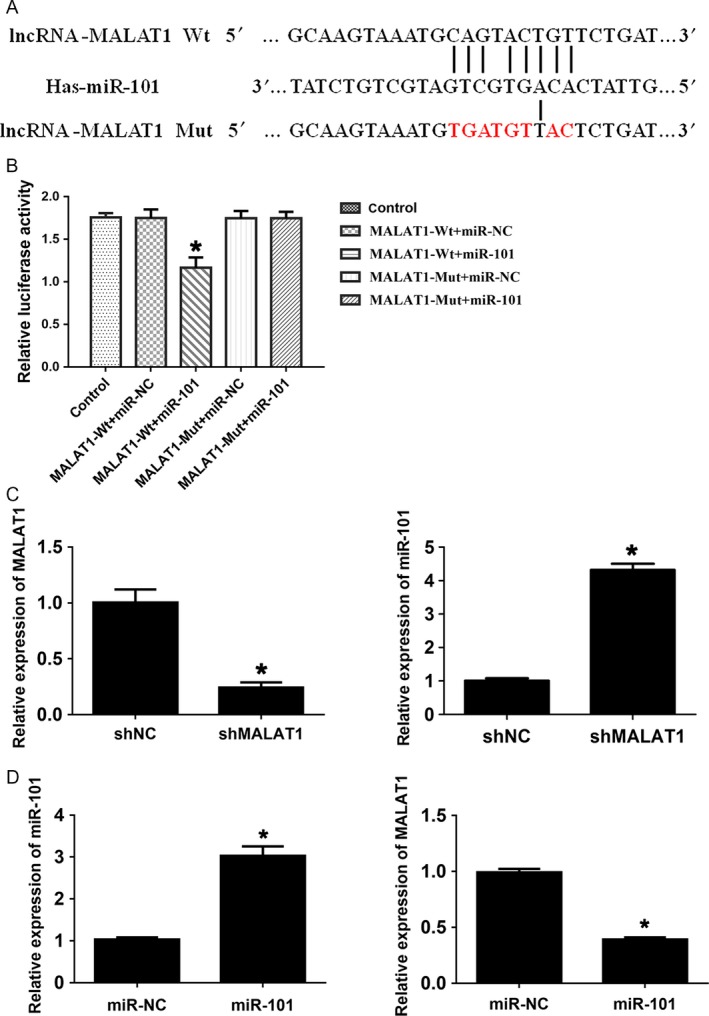
MALAT1 is a direct target of miR‐101. (A) Representation of the miR‐101 binding site in MALAT1 based on the online database microRNA.org. (B) Luciferase activity of the reporter construct containing the wild‐type or mutant miR‐101 binding site was measured after cotransfection with 50 nmol/L microRNA. (C and D) The expression of miR‐101 and MALAT1 was determined by qRT‐PCR. All experiments were performed in triplicate, *P <* *0.05.

To further investigate the potential regulatory mechanism of MALAT1 in TMZ resistance, we performed in silico analysis to identify putative interacting microRNA based on the online database microRNA.org (http://www.microRNA.org). The miR‐101 was predicted to be one of the potential interacting genes of MALAT1 (Fig. 3A). miR‐101 was found involved in sensitizing tumor to radiation and significantly downregulated in GBM cells 22. To determine whether MALAT1 is a direct target gene of miR‐101, the luciferase reporter assay was performed. These psi‐CHECK2 constructs containing putative binding site or the mutant one were cotransduced with miR‐101 mimics or miRNA NC (negative control). As shown in Figure 3B, miR‐101 repressed the luciferase activity of wild‐type MALAT1, but not that of the mutant, indicating a direct binding between MALAT1 and miR‐101. Furthermore, we assessed the expression of miR‐101 and MALAT1 in MALAT1 knockdown and miR‐101 overexpression U251 cells. Knockdown of MALAT1 significantly promoted the expression of miR‐101 in U251 cells and overexpression of miR‐101 decreased the MALAT1 expression (Fig. 3C and D). Collectively, these results indicated that MALAT1 was a direct target of miR‐101.

### Overexpression of miR‐101 reduces TMZ resistance of GBM cells

**Figure 4:**
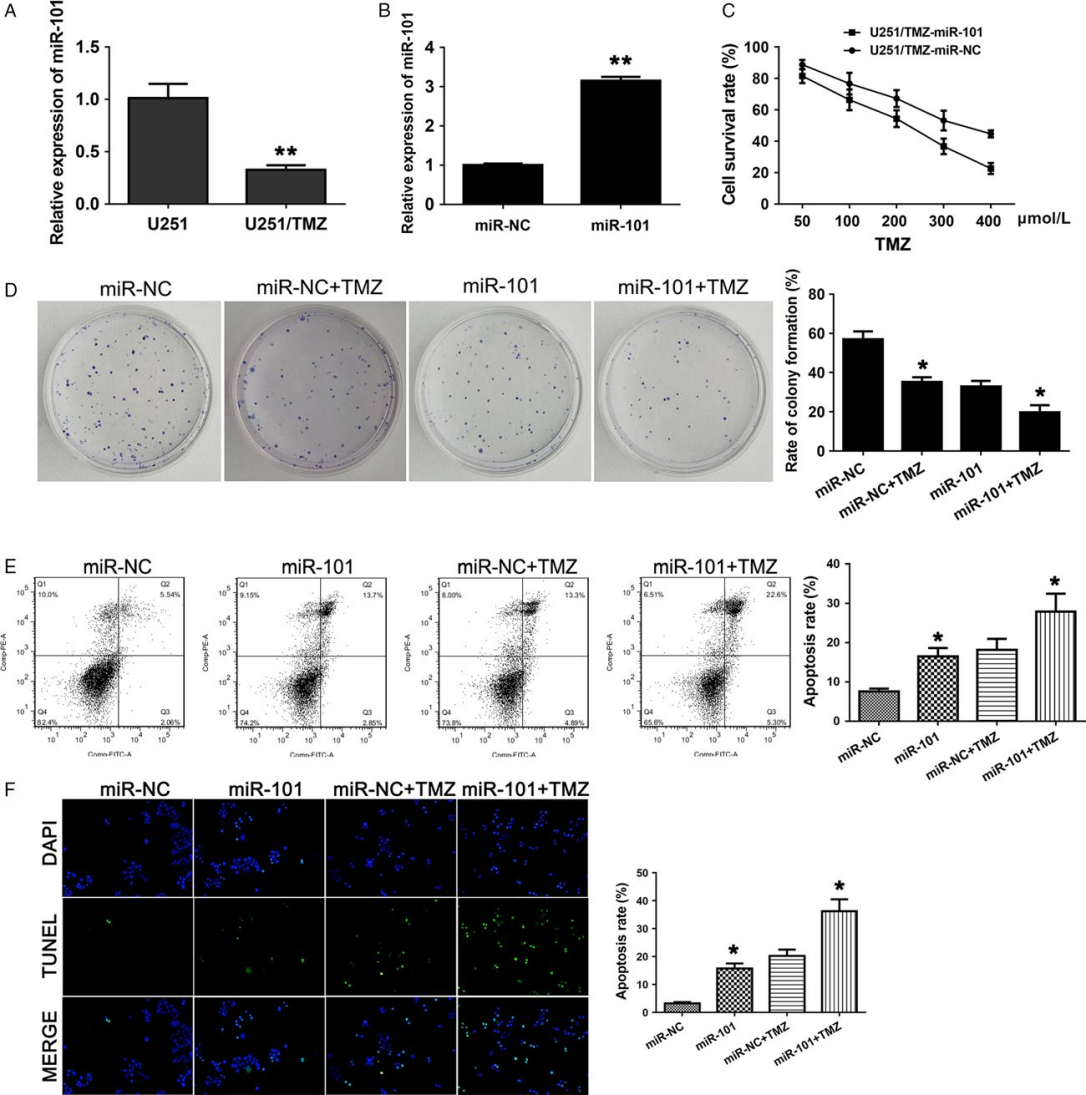
Overexpression of miR‐101 reduces TMZ resistance of GBM cells. (A) The relative expressions of miR‐101 in the U251/TMZ cells were determined by qRT‐PCR, and *β*‐actin was used as control. (B) The expressions of miR‐101 in the U251/TMZ cells with overexpression of miR‐101. (C) MTT assay was used to determine the cell survival rates of transgenic cell lines with TMZ (50–400 *μ*mol/L) treatments. (D) Colony formation assay of U251/TMZ cells transduced with miR‐101 mimics or miR‐NC in the presence or absence of TMZ. (E) Flow cytometry analysis indicated the apoptosis rates in GBM cell lines. (F) Representative DAPI‐stained nuclei and terminal deoxynucleotide transferase dUTP nick end labeling‐stained apoptotic nuclei of each group. All data represent the means ± SD of three replications. *P <* *0.05, **P <* *0.01.

To evaluate the potential role of miR‐101 in TMZ resistance, we first compared the expression of miR‐101 in U‐251/TMZ with that in its parental cell. It was shown that expression of miR‐101 largely decreased in U‐251/TMZ compared with the parental cell, suggesting high expression of miR‐101 correlated with TMZ resistance in GBM cells (Fig. 4A). Then, we overexpressed miR‐101 in TMZ‐resistant U251/TMZ cells. As shown in Figure 4B, miR‐101 in the U251/TMZ‐miR‐101 group was significantly upregulated as compared with control group. Subsequently, MTT assay was used to investigate whether overexpression of miR‐101 in U251/TMZ could influence the TMZ resistance. The U251/TMZ cells transduced with miR‐101 mimics exhibited decreased cell survival rates under TMZ treatments in comparison with the control (Fig. 4C). In addition, as indicated by colony formation assay, the high expression of miR‐101 attenuated the colony formation of TMZ‐resistant U251/TMZ cells, especially under TMZ treatment (Fig. 4D). Moreover, by flow cytometry assay and TUNEL assay, we found that the miR‐101 overexpression had a similar effect as the MALAT1 knockdown, which intensified the promotion of apoptosis rate of U251/TMZ under the treatment of TMZ in comparison with that one in the absence of TMZ treatment (Fig. 4E and F). And the results were supported by the study using GBM cell line U87 as well (Fig. S3). Taken together, these results suggest that miR‐101 and MALAT1 play antagonistic roles in regulation of TMZ resistance in GBM cells.

### MALAT1 inhibition reverses TMZ resistance by upregulating miR‐101

**Figure 5:**
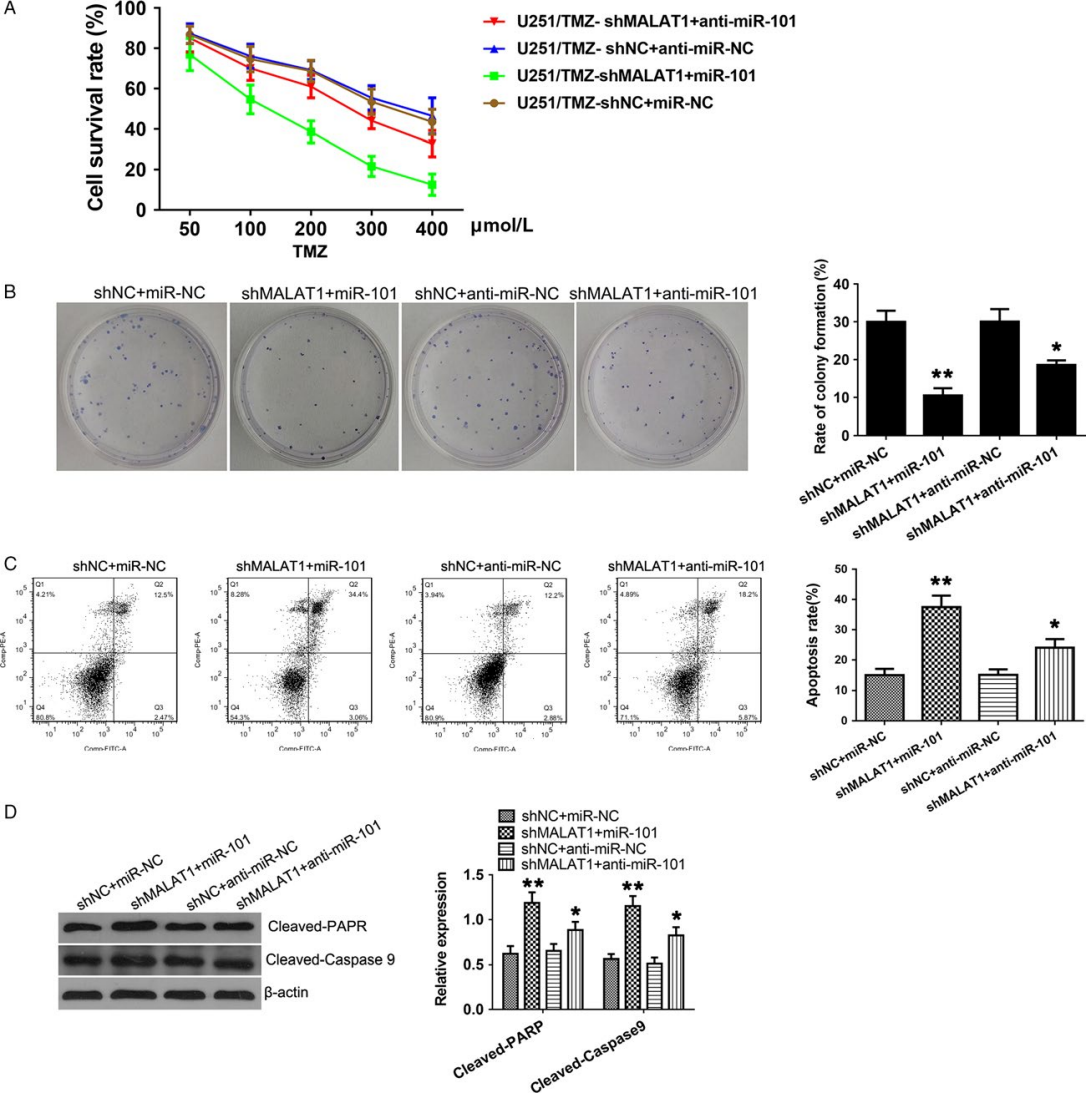
MALAT1 inhibition reverses TMZ resistance by upregulating miR‐101. (A) MTT assay was used to determine the cell survival rates of transgenic cell lines with TMZ (50–400 *μ*mol/L) treatments. (B) Colony formation assay of transgenic U251/TMZ in the presence or absence of TMZ (300 *μ*mol/L). (C) The apoptosis rate was determined by flow cytometry analysis. (D) The protein level of cleaved‐PARP and cleaved‐Caspase9 was assessed by Western blotting, and *β*‐actin was used as control. All experiments were performed in triplicate. *P <* *0.05, **P <* *0.01.

As there was a direct binding between MALAT1 and miR‐101, and they played antagonistic roles in TMZ resistance, we hypothesized that MALAT1 promotes TMZ chemoresistance thorough suppressing miR‐101. To validate this hypothesis, the U251/TMZ cell lines were cotransfected with MALAT1 knockdown vector and the miR‐101 mimics or miR‐101 inhibitors. As Figure 5A indicated, the combination of MALAT1 knockdown and miR‐101 overexpression largely reduced the chemoresistance to TMZ (50–400 *μ*mol/L). Moreover, the inhibition of TMZ resistance caused by MALAT1 knockdown was reversed by knockdown of miR‐101. Besides, colony formation assay was performed in these cotransfection groups. The combination of MALAT1 knockdown and miR‐101 overexpression caused the lowest rate of colony formation (10.53 ± 1.55%) in all cotransfection groups when were treated with 300 *μ*mol/L TMZ (Fig. 5B). In contrast, the reduction in colony formation mediated by knockdown of MALAT1 was attenuated by miR‐101 knockdown. Furthermore, as indicated by flow cytometry assay, overexpression of miR‐101 combining with knockdown of MALAT1 greatly promoted the apoptosis rate of U251/TMZ (Fig. 5C). However, knockdown of miR‐101 weakened the effect of shMALAT1 on apoptosis of U251/TMZ. In addition, we investigated the expressions of cleaved‐PARP and cleaved‐Caspase9, which were well‐known apoptosis markers 23. As shown in Figure 5D, expressions of cleaved‐PARP and cleaved‐Caspase9 were significantly elevated by the combination of MALAT1 knockdown and miR‐101 overexpression. On the contrary, the knockdown of miR‐101 attenuated the upregulations of cleaved‐PARP and cleaved‐Caspase9, which were caused by knockdown of MALAT1 in U251/TMZ. Thus, we concluded that MALAT1 inhibition reversed TMZ resistance through suppressing colony formation and promoting apoptosis by upregulating miR‐101.

### MALAT1 knockdown suppresses the expression of GSK3*β* and MGMT by upregulating miR‐101

**Figure 6:**
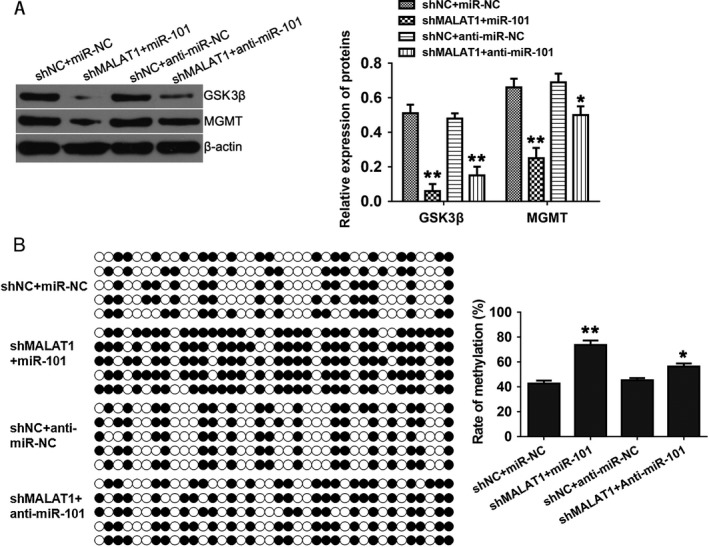
MALAT1 knockdown suppresses the expression of GSK3*β* and MGMT by upregulating miR‐101. (A) The protein levels of GSK3*β* and MGMT were determined by Western blotting, and *β*‐actin was used as control. (B) The promoter methylation of MGMT was evaluated by BSP assay. All data represent the means ± SD of three replications. *P <* *0.05, **P <* *0.01.

Previously, it was reported that miR‐101 sensitized resistant GBM cells to TMZ through downregulation of GSK3*β*, which is a multifunctional protein kinase that regulates various cellular pathways 19. Moreover, inhibition of GSK3*β* could enhance TMZ effect by decreasing O^6^‐methylguanine‐DNA methyltransferase (MGMT) expression via promoter methylation 24. To further investigate whether MALAT1 knockdown decreased TMZ resistance of GBM cells through promoting miR‐101 regulatory network, we performed the Western blotting in these transgenic lines. As shown in Figure 6A, the endogenous protein level of GSK3*β* and MGMT was significantly suppressed by combination of MALAT1 knockdown and miR‐101 overexpression. However, the suppressions of GSK3*β* and MGMT were weakened by knockdown of miR‐101. To further assess whether the suppression of MGMT was caused by the promoter methylation, we performed the bisulfite sequencing PCR (BSP) assay (Fig. 6B). The promoter methylation of MGMT was largely promoted by the combination of MALAT1 knockdown and miR‐101 overexpression, and on the contrary, knockdown of miR‐101 attenuated the upregulation of promoter methylation, which was mainly caused by knockdown of MALAT1. Taken together, these results showed that knockdown of MALAT1 could suppress the expression of GSK3*β* and MGMT by upregulating miR‐101, and these results further suggested that the influences of MALAT1 knockdown on TMZ resistance were associated with miR‐101 regulatory network.

## Discussion

As the chemoresistance is a key obstacle to the clinical outcome of chemotherapy for GBM, understanding of the molecular mechanism underlying this resistance and the identification of novel therapeutic target is urgent 25, 26, 27. MALAT1, also known as nuclear‐enriched abundant transcript 2, a highly conserved lncRNA, is dysregulated in several types of cancer, such as lung cancer, liver cancer, bladder cancer, and GBM 28, 29, 30, 31. Recently, Ma et al. 32 demonstrated that MALAT1 was significantly upregulated in glioma clinical samples and positively correlated with the malignant status and poor prognosis. However, little is known about its involvement and regulation in chemotherapy of GBM.

In the present study, we revealed that high expression of MALAT1 was associated with TMZ resistance in GBM cells. Functional studies showed that MALAT1 played important functional roles in carcinogenesis through several different mechanisms. For example, Tripathi et al. 33 reported that MALAT1 promoted cellular proliferation by controlling cell cycle progression and regulating the expression of oncogenic transcription factor B‐MYB. Additionally, Ren et al. 34 demonstrated that MALAT1 knockdown inhibited metastasis of prostate cancer and induced the cell cycle arrest. Here, our results demonstrated that MALAT1 knockdown sensitized TMZ‐resistant GBM cells to TMZ treatment by inhibiting the cell proliferation and promoting apoptosis. Importantly, the tumor formation assay in a nude mouse model demonstrated that MALAT1 had a similar effect on TMZ resistance in vivo.

It is well established that lncRNA could act as a miRNA sponge to regulate the miRNA activity by reducing the binding of miRNA to target gene. For instance, Liu et al. 35 reported that lncRNA HOTAIR promoted the proliferation, migration, and invasion of gastric carcinoma cells by sponging miR‐331‐3p and then regulating HER2 expression. In glioma cells, Cao et al. 36 demonstrated that MALAT1 suppressed the cell viability by downregulating miR‐155 and then activating FBXW7 function. In our research, we found MALAT1 containing a putative binding site for miR‐101 based on in silico analysis. With luciferase reporter assay and the gain‐ and loss‐of‐function study, we demonstrated for the first time that the direct interaction between MALAT1 and miR‐101 was crucial for regulation of TMZ resistance. Previously, Tian et al. 19 indicated that miR‐101 reversed TMZ resistance by inhibition of GSK3*β* in GBM. Moreover, the suppression of GSK3*β* could subsequently be decreasing MGMT expression via promoter methylation to regulate the TMZ resistance in GBM 24. Consistent with that study, we found GSK3*β* and MGMT were suppressed by MALAT1 knockdown and miR‐101 overexpression, which decreased the TMZ resistance in GBM, suggesting that knockdown of MALAT1 reduces TMZ resistance via promoting miR‐101 regulatory network in GBM.

In conclusion, our study demonstrates that high expression of MALAT1 is associated with TMZ resistance in GBM cells. On the other hand, MALAT1 knockdown reduces chemoresistance in TMZ‐resistant GBM cells both in vitro and in vivo and influences the cell proliferation and apoptosis. We also demonstrate that miR‐101 reduces TMZ resistance of GBM cells and plays an antagonistic role compared with MALAT1. Going one step further, we reveal that MALAT1 knockdown reverses TMZ resistance by upregulating miR‐101 regulatory network. Taken together, our findings indicate that MALAT1 may be a prognostic molecular marker and potential target for GBM TMZ‐based chemotherapy.

## Conflict of Interest

None declared.

## Supporting information


**Figure S1.** High expression of MALAT1 was associated with TMZ resistance in GBM cell U87.

**Figure S2.** Knockdown of MALAT1 reduces chemoresistance in TMZ‐resistant GBM cells in vitro and in vivo.

**Figure S3.** Overexpression of miR‐101 reduces TMZ resistance of GBM cells.
