# Supplementary figures and images for: Long noncoding RNA MALAT1 knockdown reverses chemoresistance to temozolomide via promoting microRNA‐101 in glioblastoma
Source: Cancer Med. 2018 Feb 26;7(4):1404–15. doi: 10.1002/cam4.1384 (PMC5911628; doi:10.1002/cam4.1384)

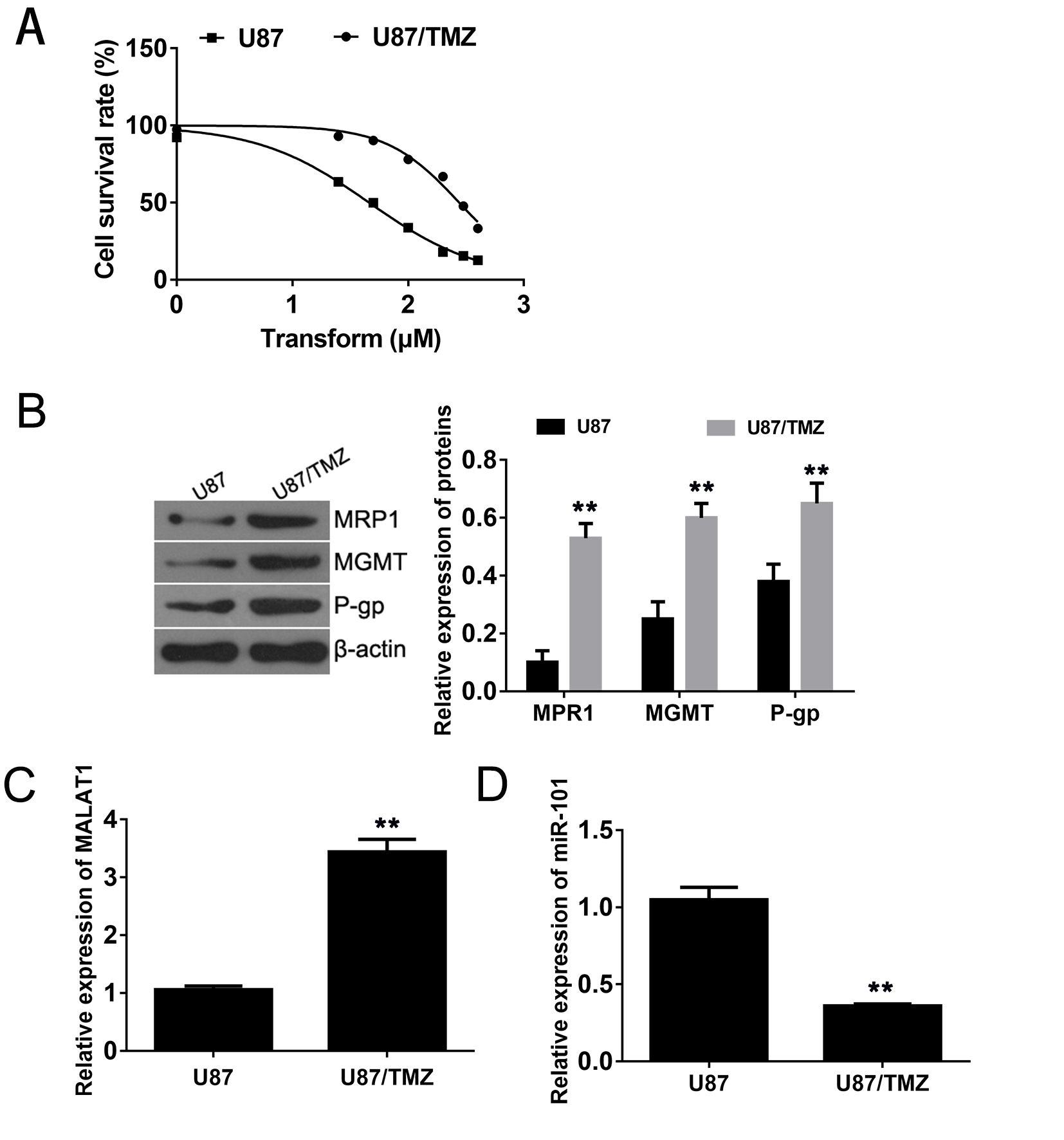

Supplement: Supplementary file 1 — Figure S1. High expression of MALAT1 was associated with TMZ resistance in GBM cell U87. [file CAM4-7-1404-s001.tif]

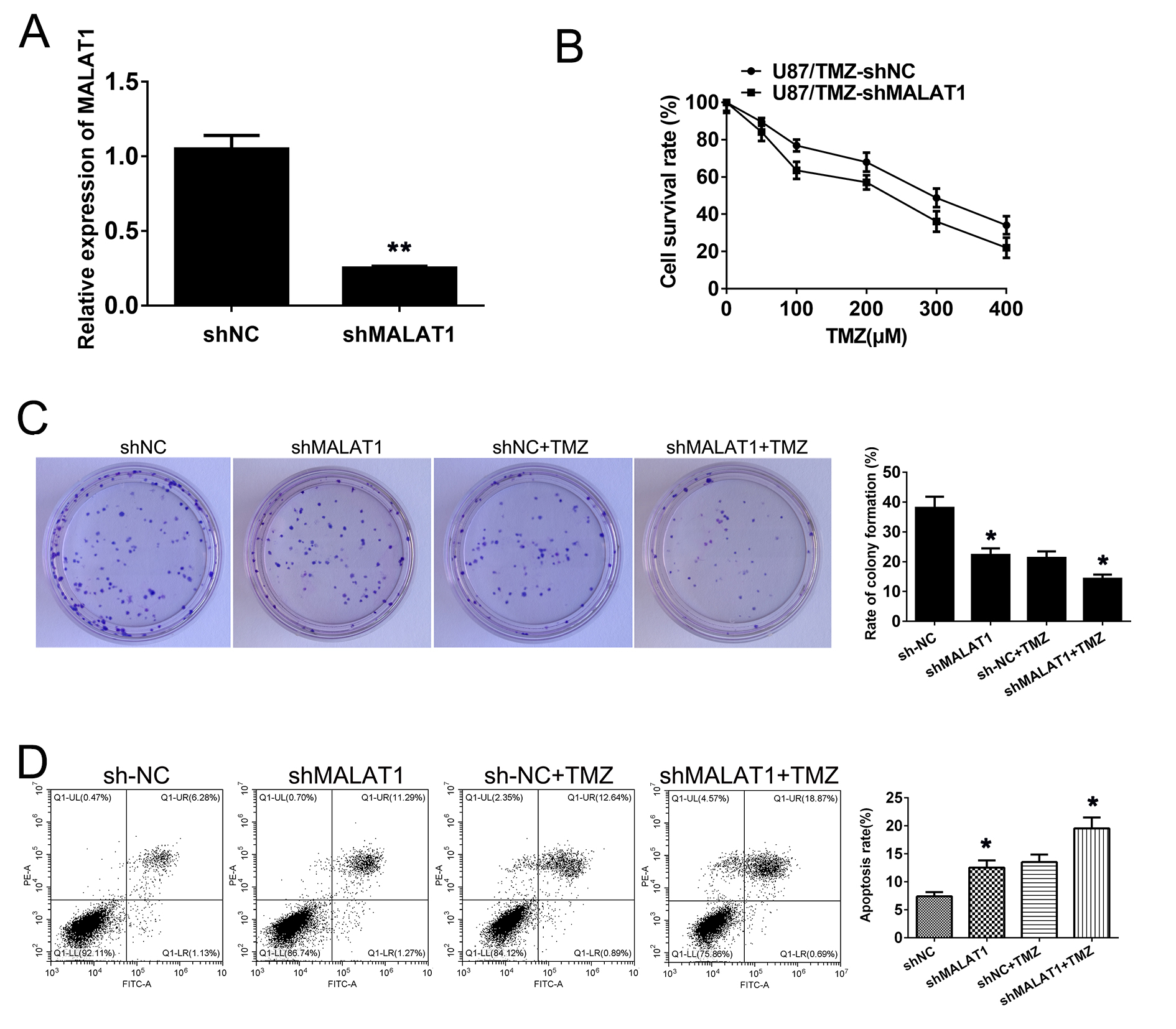

Supplement: Supplementary file 2 — Figure S2. Knockdown of MALAT1 reduces chemoresistance in TMZ‐resistant GBM cells in vitro and in vivo. [file CAM4-7-1404-s002.tif]

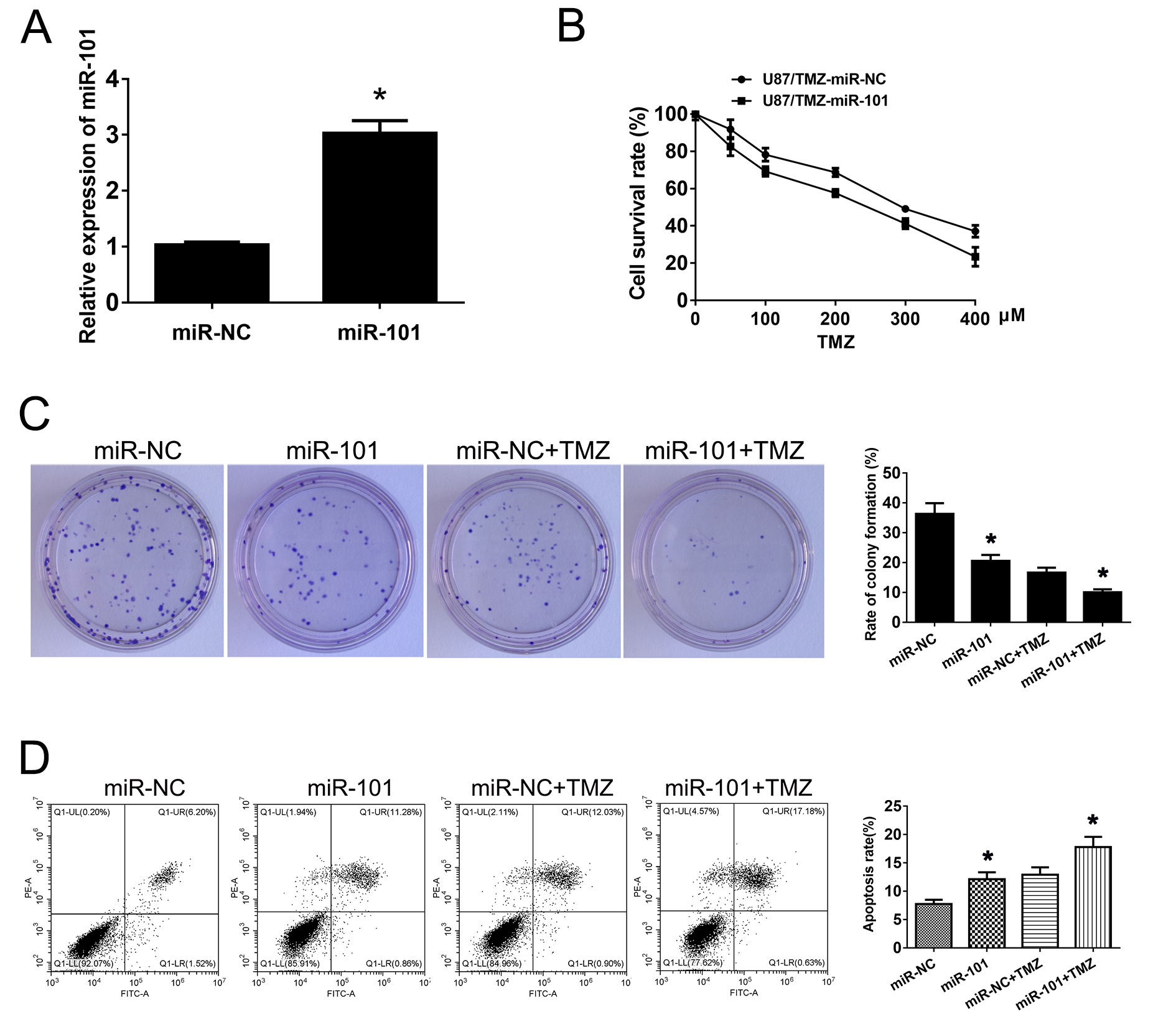

Supplement: Supplementary file 3 — Figure S3. Overexpression of miR‐101 reduces TMZ resistance of GBM cells. [file CAM4-7-1404-s003.tif]
