# Supplementary material for: Long noncoding RNA MALAT1 knockdown reverses chemoresistance to temozolomide via promoting microRNA‐101 in glioblastoma
Source: Cancer Med. 2018 Feb 26;7(4):1404–15. doi: 10.1002/cam4.1384 (PMC5911628; doi:10.1002/cam4.1384)
Supplement: Supplementary file 4 [file CAM4-7-1404-s004.docx]

**Supplemental Figure legends**

Figure S1. High expression of MALAT1 was associated with TMZ resistance in GBM cell U87. (A) Cell survival rates of GBM cell lines U87 and U87/TMZ were assessed by MTT assay. (B) The expressions of MRP1, MGMT and P-gp were evaluated by western blotting, and β-actin was used as control. (C and D) The mRNA expressions of MALAT1 and miR-101 in cell lines U87 and U87/TMZ was assessed by qRT-PCR analysis, and β-actin was used as control. ***P*<0.01.

Figure S2. Knockdown of MALAT1 reduces chemoresistance in TMZ-resistant GBM cells *in vitro* and *in vivo*. (A) The relative expressions of MALAT1 in the U87/TMZ cells transduced with shMALAT1 or shNC were determined by qRT-PCR, and β-actin was used as control. (B) The cell survival rates of transgenic cell lines with TMZ (50-400 μM) treatments were determined by MTT assay. (C) Colony formation assay showed the numbers of colonies of U87/TMZ cells transduced with shMALAT1 or shNC in the presence or absence of TMZ. (D) Annexin V/PI staining and flow cytometry analysis were used to assess apoptosis in GBM cell lines. **P*<0.05, ***P*<0.01.

Figure S3. Overexpression of miR-101 reduces TMZ resistance of GBM cells. (A) The relative expressions of miR-101 in the U87/TMZ cells were determined by qRT-PCR, and β-actin was used as control. (B) MTT assay was used to determine the cell survival rates of transgenic cell lines with TMZ (50-400 μM) treatments. (C) Colony formation assay of U87/TMZ cells transduced with miR-101 mimics or miR-NC in the presence or absence of TMZ. (D) Annexin V/PI staining and flow cytometry analysis were used to assess apoptosis in GBM cell lines. **P*<0.05, ***P*<0.01.
